# Adequate exposure of 50 mg dolutegravir in children weighing 20 to 40 kg outside of sub-Sahara Africa

**DOI:** 10.1097/QAD.0000000000003350

**Published:** 2022-09-15

**Authors:** Hylke Waalewijn, Kim Stol, Linda van der Knaap, Pieter L.A. Fraaij, Clementien Vermont, Annemarie M.C. van Rossum, Riet Strik-Albers, David M. Burger, Elin M. Svensson, Angela Colbers

**Affiliations:** a Radboud University Medical Center, Department of Pharmacy, Radboud Institute for Health Sciences (RIHS), Nijmegen, The Netherlands; b Radboud University Medical Center, Amalia Children's Hospital, Department of Pediatrics, Division of Infectious Diseases and Immunology, Nijmegen, The Netherlands; c Erasmus MC University Medical Center-Sophia Children's Hospital, Department of Pediatrics, Division of Infectious Diseases and Immunology, Rotterdam, The Netherlands; d Department of Viroscience, Erasmus-MC University Medical Center, Rotterdam, The Netherlands; e Department of Medical Microbiology and Infectious Diseases, Erasmus MC University Medical Center, Rotterdam, The Netherlands; f Department of Pharmacy, Uppsala University, Uppsala, Sweden.

## Abstract

Dolutegravir 50 mg is registered for use in children weighing 20–40 kg. This approval is based on data from an African paediatric cohort, and no pharmacokinetic data was available from children outside of Africa. This study provides further evidence of the effective use of dolutegravir 50 mg in children weighing 20 to 40 kg by showing that concentration data gathered in clinical practice shows adequate concentration levels in Dutch children without a safety signal.

The viral integrase enzyme inhibitor dolutegravir is currently used as a first-choice antiretroviral for treating HIV infection in adults and children. However, dolutegravir treatment was inaccessible for most children living with HIV because the 50 mg film-coated tablet registered for use in adults and adolescents weighing >40 kg, was the only formulation available in sub-Saharan Africa. This changed when data from the ODYSSEY trial showed that 50 mg dolutegravir, taken by children weighing more than 20 kg, achieved drug exposures comparable to adult reference values [1]. Based on these data, in 2020, the FDA and European Medicines Agency have adopted this dose change [2,3].

We investigate whether this dose would also achieve appropriate dolutegravir exposure in children outside of Africa. We study plasma concentration data gathered in routine clinical practice in children weighing 20–40 kg treated with dolutegravir 50 mg once-daily in the Netherlands.

This was a retrospective multicentre pharmacokinetic study, investigating dolutegravir concentration data, which was collected from children who gave written informed consent (or caretaker consented), weighing 20–40 kg, who had been on antiretroviral treatment for at least 1 year. The study was reviewed by the relevant ethics review committees (METC no.: 2019-6039), and approved as a retrospective study not interfering with clinical practice.

The primary objective of the study was to compare the trough concentration (*C*_trough_) of 50 mg dolutegravir administered to children with HIV in the Netherlands weighing 20–40 kg to *C*_trough_ values of adults taking 50 mg dolutegravir with food (1.11 mg/l) [3], and *C*_trough_ data from the ODYSSEY trial of children weighing 20–40 kg taking dolutegravir without food (0.72 mg/l) [1]. In addition, safety and efficacy data was explored through a retrospective review of clinical records. Individual *C*_trough_ predictions and the proportion of samples below the 90% effective concentration (EC_90_: 0.32 mg/l) were reported [4].

Dolutegravir plasma concentration data was gathered according to local drug-monitoring practices, recording at least the dose, time and date of the last taken dose, co-medication, and time and date of the therapeutic drug monitoring (TDM) sample as well as weight and height of the child. All samples were measured with a validated analysis method [5]. All grade 3 and 4 adverse events (AE), serious adverse events (SAE), and any event resulting in discontinuation of dolutegravir were reported. Viral blip is defined as a single viral load between 40 and 200 copies/ml followed by viral suppression. Missing information on patients’ weight was handled by taking the weight of a visit within 4 weeks of the sampling visit or by assuming linear weight change between two visits.

Plasma samples were taken during scheduled periodic clinic visits at arbitrary time after a self-reported dolutegravir dose. These samples were used to predict the concentration at 24 h after dose (*C*_trough_) using a paediatric pharmacokinetic population model in NONMEM (see model control stream, Supplementary Digital Content 1, which describes the model control stream of the population-pharmacokinetic model) [6,7]. This method was accurate and precise for the purpose of our analysis (see model validation section, Supplementary Digital Content 2, which shows the accuracy and precision of the used method).

Children could have multiple samples taken at different occasions on the same dose. To prevent giving more weight to children that had TDM samples at multiple occasions, a single geometric mean (GM) *C*_trough_ for each individual was calculated by combining predicted *C*_trough_ from the available samples. GM *C*_trough_ from individuals were combined in a GM *C*_trough_ for the cohort.

In total, 20 participants were enrolled in this study (Table 1 for demographics information). In total, 76 TDM visits were included in the analysis. Children contributed a median (range) of four (1–9) samples with six children contributing only one sample (see Figure 1, Supplementary Digital Content 3, which shows distribution of sample data in time after dose).

**Table 1:** Demographics and trough concentration results of children taking 50 mg dolutegravir.

Dose (as film-coated tablet)	50 mg
Number of children with TDM samples on dose (female)	20 (13)
Ethnicity Black African; mixed Black African-white	18; 2
Means of infection (MTCT; unknown)	9; 11
Total plasma concentration samples	76
Weight (kg)	30.65 (27.68–34.06)
Age (years) [IQR; range]	10.0 (10.0–10.25; 5.9–13.8)
The median (range) follow-up^∗^	1.65 (0–2.07) years
Total follow-up time	23.8 treatment years
DTG *C*_trough_ (mg/l)^∗∗^	1.93 (32)
Reference *C*_trough_ fasted children (mg/l)^†^	0.72 (44)
Reference *C*_trough_ fed adults (mg/l)^¥^	1.11 (46)
Individuals with viral blip	2/20

Median (IQR) for weight and age and geometric mean *C*_trough_ (CV%); *C*_trough_: concentration at the end of dosing interval (*T* = 24 h); MTCT: mother to child transmission. For our study this concentration is extrapolated to *T* = 24 h from a TDM sample. ^∗^Time between the first TDM sample and last data entry. ^∗∗^Mostly without regards to food. ^†^Calculated from fasted children receiving 50 mg in the ODYSSEY trial. ^¥^Fed adults [3].

GM *C*_trough_ with coefficient of variation (CV%) was 1.93 (32) mg/l, which is higher than reference values: 1.1 mg/l of adults taking dolutegravir 50 mg once-daily with food, and 0.72 of children weighing 20–40 kg in the ODYSSEY trial, taking dolutegravir without food (Table 1). Two of 76 (2.6%) of individual estimated *C*_trough_ were below dolutegravir EC_90_ of 0.32 mg/l. One was from a child with a history of nonadherence to therapy.

Two out of 20 children had a detectable viral load >40 copies/ml in the period of observation: one blip of 40 copies/mL followed by viral suppression after 1 month and one child, with a history of non-adherence to therapy, remained detectable during 6 months of 50 mg dolutegravir treatment. There was no apparent relation to measured *C*_trough_ and the child continued to be suppressed from 8 months after start of therapy and onwards. All other children had an undetectable viral load during the studied period. No dolutegravir doses where changed based on plasma concentrations.

One AE resulted in dolutegravir discontinuation: nocturnal nose bleeds combined with loss of concentration, possibly related to dolutegravir use (GM *C*_trough_: 0.4 mg/l). One SAE was reported: increased lipid and amylase levels, attributed to lopinavir/ritonavir use. Lipid and amylase levels normalized after stopping lopinavir/ritonavir. Dolutegravir was continued without further adverse events.

We report adequate exposure in children 20–40 kg, living in the Netherlands, receiving 50 mg dolutegravir once-daily. GM *C*_trough_ was higher than DTG reference values established in adults and children, without concerning safety issues in our observational period (Table 1). The reference paediatric cohort took dolutegravir without food, while, in our study, almost all children took dolutegravir with food. In adults, food intake increases dolutegravir exposure by 70% [7]. The difference in food intake and nutritional status of the two paediatric cohorts potentially explains the difference between the studies.

No relation between *C*_trough_ parameters and toxicity has been reported in the literature and paediatric studies have confirmed long-term safety of dolutegravir in children [8,9]. Moreover, GM *C*_trough_ of 2.12 mg/l was safe in adults receiving 50 mg dolutegravir twice-daily [3]. The low number of adverse events related to dolutegravir in our study suggests that these exposures remain safe.

In conclusion, 50 mg dolutegravir provides adequate exposure to dolutegravir in children above 20 kg, living in the Netherlands. We report the first pharmacokinetic data on 50 mg dolutegravir used clinically in children weighing 20–40 kg outside of sub-Sahara Africa and provide confirmation that the 50 mg dolutegravir dose provides adequate exposure and was well tolerated when taken by children in different settings.

## Acknowledgements

No funding was received for this study.

### Conflicts of interest

There are no conflicts of interest.

## Supplementary Material

Supplemental Digital Content

## References

[1] Bollen PDJ Moore CL Mujuru HA Makumbi S Kekitiinwa AR Kaudha E. Simplified dolutegravir dosing for children with HIV weighing 20 kg or more: pharmacokinetic and safety substudies of the multicentre, randomised ODYSSEY trial. *Lancet HIV* 2020; 7:e533–e544. 32763217 10.1016/S2352-3018(20)30189-2 PMC7445428

[2] European Medicines Agency. Tivicay: dolutegravir. https://www.ema.europa.eu/en/medicines/human/EPAR/tivicay [Accessed 5 February 2022].

[3] US Food and Drug Administration. New Drug Application (NDA): 213983. Products on NDA 213983: Tivicay PD. https://www.accessdata.fda.gov/scripts/cder/daf/index.cfm?event=overview. process&ApplNo=213983 [accessed 22 April 2022]

[4] Min S Sloan L DeJesus E Hawkins T McCurdy L Song I. Antiviral activity, safety, and pharmacokinetics/pharmacodynamics of dolutegravir as 10-day monotherapy in HIV-1-infected adults. *AIDS* 2011; 25:1737–1745. 21716073 10.1097/QAD.0b013e32834a1dd9

[5] Bollen PDJ de Graaff-Teulen MJA Schalkwijk S van Erp NP Burger DM. Development and validation of an UPLC-MS/MS bioanalytical method for simultaneous quantification of the antiretroviral drugs dolutegravir, elvitegravir, raltegravir, nevirapine and etravirine in human plasma. *J Chromatogr B Analyt Technol Biomed Life Sci* 2019; 1105:76–84. 10.1016/j.jchromb.2018.12.008 30572204

[6] Singh R, Baker M, Thapar M, Gibb D, Turkova A, Ford D, *et al.* **Pediatric dolutegravir (DTG) dosing recommendations derived from combined P1093 and ODYSSEY Population Pharmacokinetic analysis**. In: *International Workshop on HIV & Pediatrics 2020*. San Francisco; 2020.

[7] US Food and Drug Administration. **Clinical pharmacology and biopharmaceutics review(s)**. https://www.accessdata.fda.gov/drugsatfda_docs/nda/2020/213983Orig1s000ClinPharmR.pdf [Accessed 5 February 2022].

[8] Lugemwa A, Mujuru H, Wynne B, Violari A, Kekitiinwa A, Kity C, et al. **A randomised comparison of DTG-based ART vs standard of care in infants and young children living with HIV weighing 3 to 14** **kg: results from the ODYSSEY trial**. [conference proceeding PEBLB18] In: International AIDS society (IAS) 18–21 July 2021.

[9] Turkova A White E Mujuru HA Kekitiinwa AR Kityo CM Violari A. Dolutegravir as first- or second-line treatment for HIV-1 infection in children. *N Engl J Med* 2021; 385:2531–2543. 34965338 10.1056/NEJMoa2108793 PMC7614690

