# Supplementary material for: Adequate exposure of 50 mg dolutegravir in children weighing 20 to 40 kg outside of sub-Sahara Africa
Source: AIDS. 2022 Sep 15;36(14):2077–9. doi: 10.1097/QAD.0000000000003350 (PMC9612713; doi:10.1097/QAD.0000000000003350)
Supplement: Supplemental Digital Content [file aids-36-2077-s001.docx]

Adequate exposure of 50mg dolutegravir in children weighing 20 to 40 kg outside of Sub-Sahara Africa

MSc Hylke Waalewijn^1^, Dr Kim Stol^2,3^, MANP Linda van der Knaap^3^, Dr Pieter L.A Fraaij^3,4^, Dr Clementien Vermont, Prof Dr Annemarie M.C. van Rossum^3,5^, MANP RN Riet Strik-Albers^2^, Prof Dr David M Burger^1^, Dr Elin M Svensson^1,6^, Dr Angela Colbers^1^

# Content

1: Model control stream

2: Additional data

2: Method validation

## 1 Model control stream[1]

$SIZES LVR = 37

$PROB SIM DTG

$INPUT C ID TIME DV EVID MDV OCC AMT SS II CMT WT FASTED AGE WB

$DATA data.csv IGNORE(C.EQ.C)

$SUBROUTINES ADVAN5

$MODEL NCOMP=2

COMP = (DEPOT)

COMP = (CENTRAL) ; ALL OBSERVATIONS IN CMT=2

$PK

PMA=AGE*52+40 ; AGE (YEARS) NEEDS TO BE A COLUMN IN DATASET

HILL=3.43

TM50=52.2

FORM = 0

FMAT=(PMA**HILL/(PMA**HILL+TM50**HILL))

ALLOCL=(WT/70)**0.455

ALLOV=(WT/70)**0.556

IF (OCC.EQ.1) IOVCL=ETA(4) ;

… ; fill to 16 occasions

IF (OCC.EQ.16) IOVCL=ETA(19) ;

IF (OCC.EQ.1) IOVKA=ETA(20) ;

… ; fill to 16 occasions

IF (OCC.EQ.16) IOVKA=ETA(35) ;

CL=THETA(1)*FMAT*ALLOCL*EXP(ETA(1))*EXP(IOVCL)

V=THETA(2)*ALLOV*EXP(ETA(2))

IF (FORM.EQ.0) KA=THETA(3)*EXP(ETA(3)) ; FCT – formulation (0 OR 1)

IF (FORM.EQ.1) KA=THETA(4)*EXP(ETA(3)) ; GRANULES

IF (FORM.EQ.0) F1=1*((1.1)**FASTED) ;FASTING (0 IS WITHOUT REGARD TO FOOD OR 1 IS FASTED)

IF (FORM.EQ.1) F1=1.53

S2=V

K12=KA

K20=CL/V

$ERROR

IPRED=F

Y=IPRED+(IPRED*ERR(1))+ERR(2)

$THETA

1.03; 1 CL

13.6 ; 2 V

0.854 ; 3 KA FCT

1.74; 4 KA GRANULES

$OMEGA BLOCK(3)

0.0863 ;1 CL

0.0499 0.0698; 2 CL-V V

0.0953 0.138 0.762; 3 CL-KA, V-KA, KA

$OMEGA BLOCK(1) 0.115 ; 4 IOV CL ;

… ; fill to 16 occasions

$OMEGA BLOCK(1) SAME ; 19 ;

$OMEGA BLOCK(1) 0.610 ; IOV KA ;

… ; fill to 16 occasions

$OMEGA BLOCK(1) SAME ; 35 ;

$SIGMA

0.0123 ; PROP ERR

0.09 ; ADD ERR IN MG/L

$EST METHOD=1 INTERACTION MAXEVAL=0 POSTHOC NSIG=3 SIGL=9

$TABLE ID TIME OCC DV AMT MDV EVID SS II FASTED AGE WT FORM WB IPRED ONEHEADER NOPRINT FILE=PIXEL_sdtab.tab

$TABLE ID CL V KA IPRED CWRES NPDE ETAS(1:LAST) ESAMPLE=1000 ONEHEADER NOPRINT FILE=PIXEL_patab.tab

## Additional data


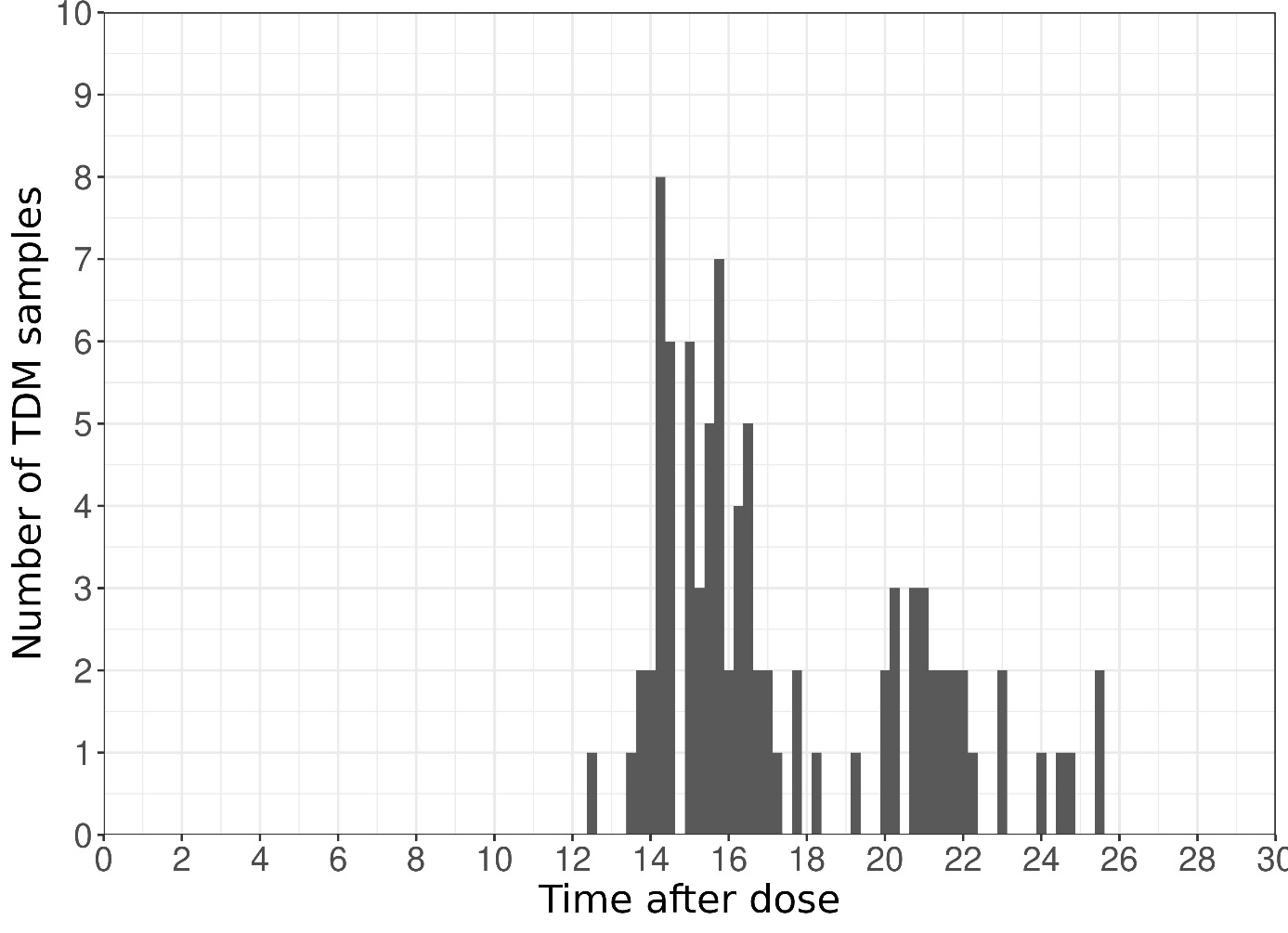


Supplementary figure 1. Time after dose of TDM samples that were analysed showing majority of samples being taken around 14 hours after dose.


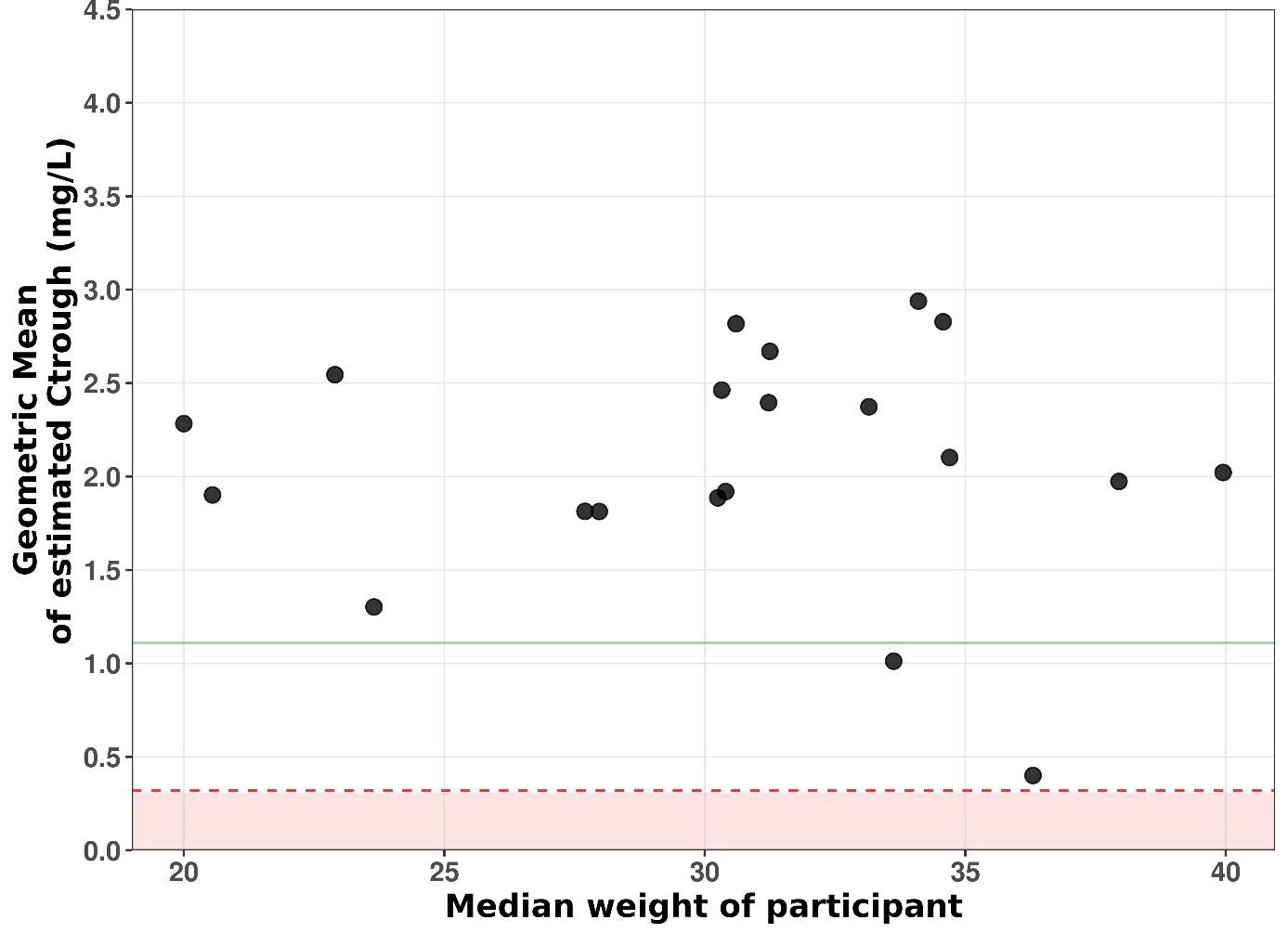


Supplementary figure 2. Geometric mean of estimated trough concentrations (C_trough_) with median weight at time of TDM samples per participant. Horizontal dashed line indicates EC_90_ value of 0.32mg/L, the solid line represents the geometric mean C_trough_ of adults on 50mg dolutegravir once-daily who took dolutegravir with food.

### **Method validation**

#### Comparison between re-estimated C_trough_ and simulated (real) C_trough_:

The goal of our analysis was to estimate C_trough_ from single TDM samples using a population PK model developed based on a pediatric population of children >4 weeks old together with dose data and patient characteristics. For the method validation we used a virtual pediatric population based on HIV infected children and added the relevant covariates fasting status and formulation type (film coated table; FCT) to create a simulation dataset [2].


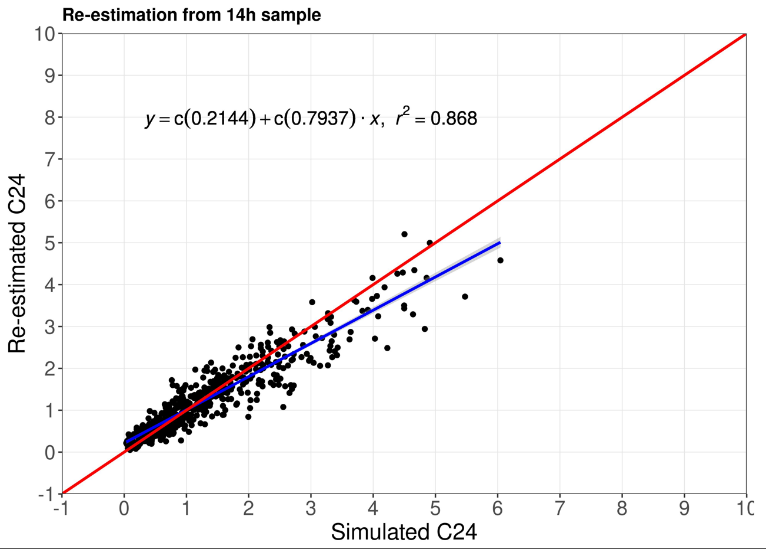

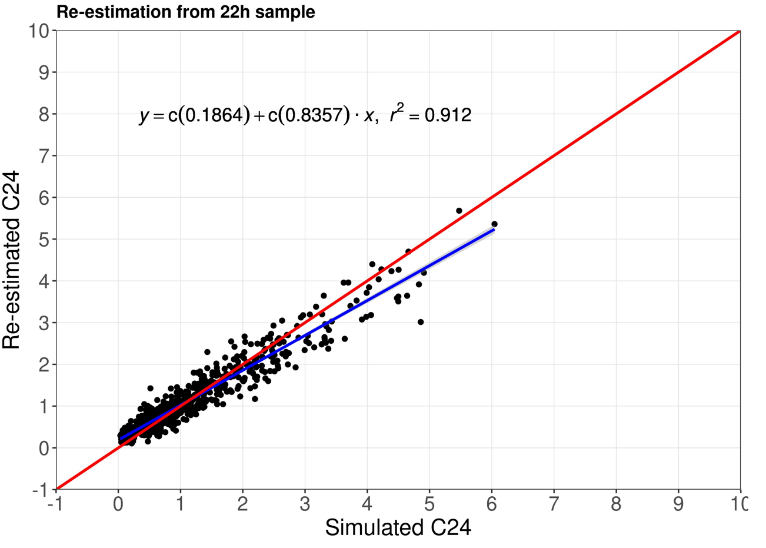
With the population dataset and the pediatric dolutegravir model we simulated C_trough_ data and concentration data at timepoints 14 and 22 hours after dose. We chose these timepoints based on the observed spread of the TDM samples in our study (supplementary figure 1). We evaluated the ability of the model to re-estimate the C_trough_ using a single TDM sample to replicate the study’s situation. The figures below show that there is some under estimation of C_trough_ at high C_trough_ concentrations with our method. The median (IQR) trough concentration in our study is 1.90(1.27-2.56) mg/L. At these concentrations the estimated C_trough_ from samples taken 14 and 22 hours after dose correlate well with simulated C_trough_. We therefore have concluded that underestimation would likely be minimal in our study and that our method is therefore suitable for the purpose of our study.

Supplementary figure 3. Simulated C_trough_ versus re-estimated C_trough_ from samples at T=14h (left) and T=22h (right) after dose using the model. Indicating our method is suitable to be used for this application.

#### C_trough_ estimation Bootstrap

We performed a bootstrap analysis (10000 repetitions) to evaluate the accuracy and precision of estimating a geometric mean based on 15 samples. We chose 15 samples to evaluate our method under more strict conditions than conditions in our study where we had data from 20 children with C_trough_ data from multiple samples. The below histograms are the resulting frequencies of estimated GMs for simulated samples of children taking 50mg DTG with samples taken at 14 and 22 hours after dose.

The histograms show a wide variation of GM but distributions do seem to be centred around the true GM. Median with 90% confidence intervals for the bootstrap histograms are 0.96 (0.88-1.08) and 0.99 (0.90-1.13) for C_trough_ extrapolated from 14 hours and 22 hours after dose, respectively. This matches closely with the simulated (true) C_trough_ of 0.95. We have therefore concluded that our method is sufficiently accurate and precise for the purpose of our study.


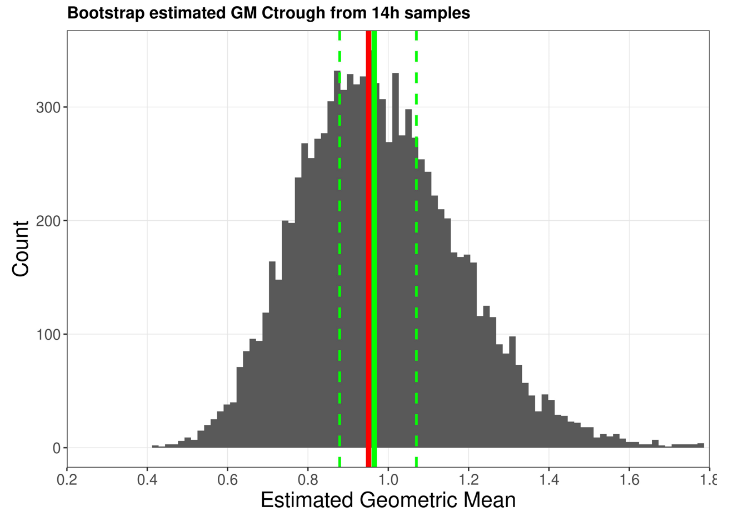

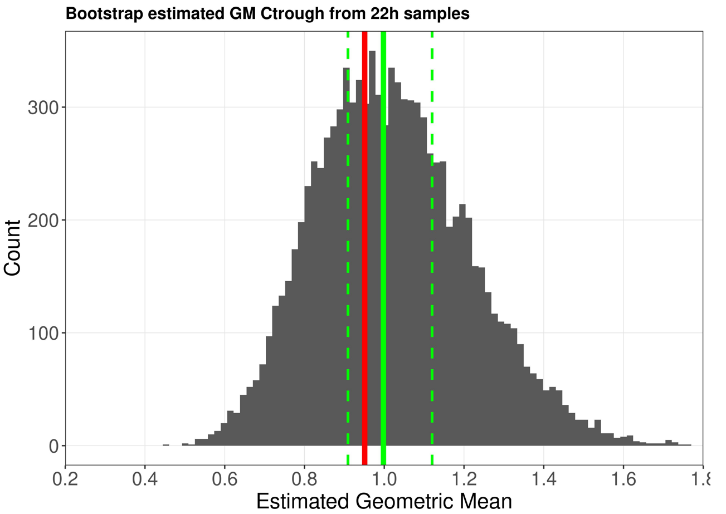


Supplementary figure 4. Histograms are the resulting frequencies in GMs for estimated samples of children taking 50mg DTG with samples taken 14 hours after dose (left) and 22 hours after dose (right). The red line represents the true GM C_trough_ for the simulated children, while the green solid line is the median estimated GM C_trough_ value with 90% confidence interval in green dashed lines.

1. FDA, *Tivicay PD, Clinical Pharmacology and Biopharmaceutics Review(s)*, U. FDA, Editor.: Drugs@FDA.

2. Wasmann, R.E., et al., *Constructing a representative in-silico population for paediatric simulations: Application to HIV-positive African children.* Br J Clin Pharmacol, 2021. **87**(7): p. 2847-2854.
